# The effect of permodified cyclodextrins encapsulation on the photophysical properties of a polyfluorene with randomly distributed electron-donor and rotaxane electron-acceptor units

**DOI:** 10.3762/bjoc.10.222

**Published:** 2014-09-09

**Authors:** Aurica Farcas, Ana-Maria Resmerita, Pierre-Henri Aubert, Flavian Farcas, Iuliana Stoica, Anton Airinei

**Affiliations:** 1 Inorganic Polymers, ‘‘Petru Poni’’ Institute of Macromolecular Chemistry, Grigore Ghica Voda Alley, 700487 Iasi, Romania; 2 Laboratoire de Physicochimie des Polymères et des Interfaces (EA 2528), Institut des Matériaux, Université de Cergy-Pontoise, F-95031 Cergy-Pontoise Cedex, France; 3 “Gh. Asachi” Technical University, 61–63 Mangeron Blvd, 700050 Iasi, Romania

**Keywords:** cyclodextrins, energy band gaps, fluorescence lifetimes, persilylated cyclodextrins, supramolecular encapsulation, surface morphology

## Abstract

We report on the synthesis as well as the optical, electrochemical and morphological properties of two polyrotaxanes (**4a** and **4b**), which consist of electron-accepting 9,9-dicyanomethylenefluorene **1** as an inclusion complex in persilylated β- or γ-cyclodextrin (TMS-β-CD, TMS-γ-CD) (**1a**, **1b**) and methyltriphenylamine as an electron-donating molecule. They are statistically distributed into the conjugated chains of 9,9-dioctylfluorene **3** and compared with those of the corresponding non-rotaxane **4** counterpart. Rotaxane formation results in improvements of the solubility, the thermal stability, and the photophysical properties. Polyrotaxanes **4a** and **4b** exhibited slightly red-shifted absorption bands with respect to the non-rotaxane **4** counterpart. The fluorescence lifetimes of polyrotaxanes follow a mono-exponential decay with a value of τ = 1.14 ns compared with the non-rotaxane, where a bi-exponential decay composed of a main component with a relative short time of τ_1_ = 0.88 (57.08%) and a minor component with a longer lifetime of τ_2_ = 1.56 ns (42.92%) were determined. The optical and electrochemical band gaps (Δ*E*_g_) as well as the ionization potential and electronic affinity characterized by smaller values compared to the values of any of the constituents. AFM reveals that the film surface of **4a** and **4b** displays a granular morphology with a lower dispersity supported by a smaller roughness exponent compared with the non-rotaxane counterpart.

## Introduction

Semiconducting π-conjugated polymers have attracted attention in the last years as promising active hole-transporting materials, which have a wide range of applications in electro-optical devices [1–4]. Polyfluorenes (PFs) are the most often investigated semiconducting polymers and are considered promising candidate for flexible displays and blue light-emitting materials [5]. Unfortunately, PFs exhibit higher electrochemical band gaps and unbalanced charge injection. Moreover, they are characterized by the tendency to form aggregates/excimers or keto defects in the solid state upon device operation [6]. A variety of approaches were examined, e.g., copolymerization [7–11], end-capping with sterically hindered groups [12], introduction of donor (D) and acceptor (A) moieties, to form statistically [13–14] or alternating D–A units in the main chain [15–18]. The coupling of D–A units has been used to synthesize copolymers with smaller electrochemical and optical band gaps, so that materials with improved electronic and optical properties can be obtained [17]. The construction of mechanically interlocked molecules such as rotaxanes and polyrotaxanes with native cyclodextrins (CDs) as macrocycle molecules has been employed as an alternative approach to achieve the control of aggregation and to improve the photophysical and morphological characteristics [13]. In the past years, it has been demonstrated that the encapsulation of conjugated polymer into macrocycle cavities leads to an “insulation” of the individual molecular wires. Moreover, recently reported results have shown improvements of the thermal stability, solubility, luminescence and surface characteristics of polyrotaxanes compared to those of non-rotaxane counterparts [8,13,19–25]. This approach has also been applied for the synthesis of polyrotaxanes by incorporating chemically modified CDs as host molecules [11,26–30]. Due to lower tendency to aggregate formations of functionalized CDs [31], polyrotaxanes with permodified CDs are characterized by a better solubility in common organic solvents, a higher fluorescence efficiency, a higher transparency of the solid films, and an easier processability compared with native CDs. These improvements represent noticeable advantages of conjugated polyrotaxanes for optoelectronic applications. The combination of D–A units associated with the encapsulation of an acceptor monomer into CDs has been successfully employed for the fabrication of PFs materials with smaller optical and electrochemical band gaps [13].

**Figure 1:**
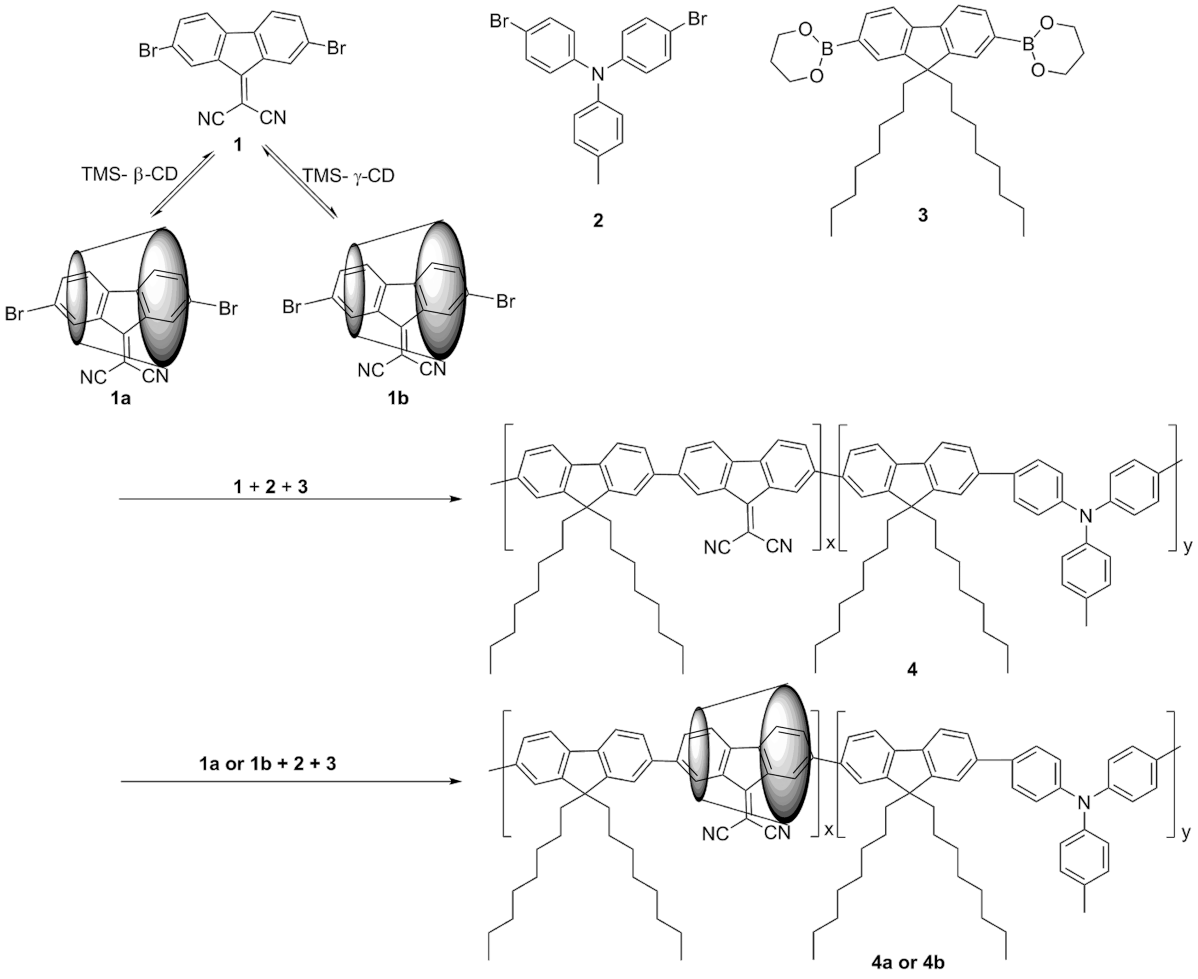
Top: Chemical structures of the starting materials. Bottom: Synthetic route of the non-rotaxane **4**, and polyrotaxanes **4a** and **4b** with TMS-β-CD and TMS-γ-CD, respectively.

Herein, we report on the synthesis as well as the characterization of the optical, electrochemical and morphological properties of **4a** and **4b** polyrotaxanes, which consist of **1a** or **1b** and **2** units statistically distributed into the conjugated chains of **3** and compared them with those of the corresponding **4** non-rotaxane counterpart (Figure 1).

## Results and Discussion

To explore the effect of TMS-β-CD and TMS-γ-CD encapsulations toward native γ-CD on photophysical properties of PFs we performed the present study. TMS-β-CD and TMS-γ-CD macrocyclic molecules, **1** and **2** monomers, were prepared according to previously reported procedures [13,32–35]. The chemical structure of completely persilylated compounds was confirmed by using FTIR and NMR spectroscopy (see details in Figures S1 and S2 in Supporting Information File 1).

**Figure 2:**
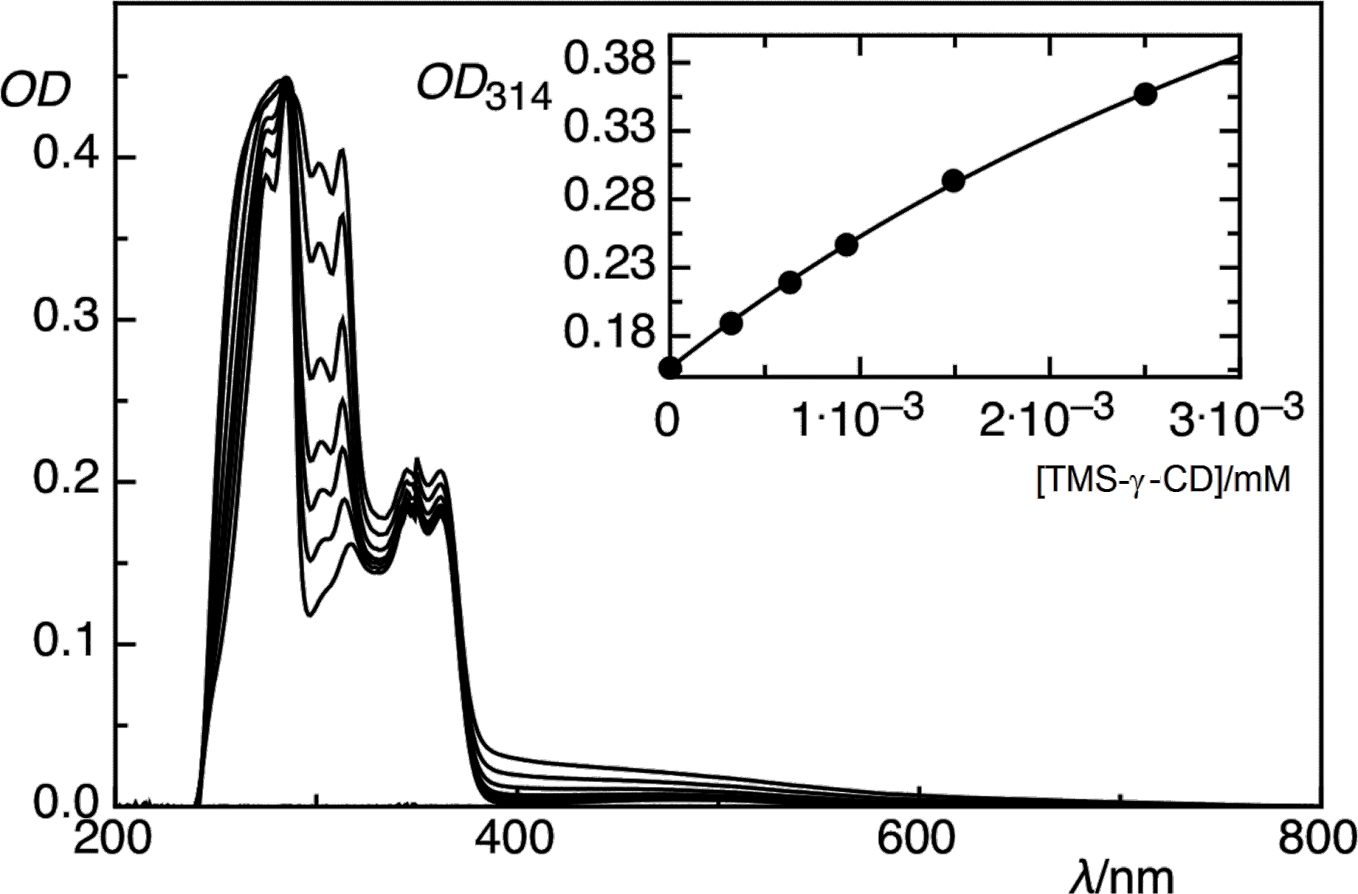
Changes in the absorption spectra of the monomer **1** upon the addition of increasing amounts of TMS-γ-CD in CHCl_3_. The inset shows the fitted binding constant curve based on a 1:1 host–guest complexation stoichiometry.

To clarify whether the guest molecule **1** can be accommodated inside the permodified CDs cavities, the approximated stability constant (*K*_s_) was investigated. The *K*_s_ of **1a** and **1b** inclusion complexes in toluene cannot be measured accurately due to the strong UV absorbance of this solvent. Thus, *K*_s_ measurements were performed in CHCl_3_. The *K*_s_ was determined by measuring the change in the absolute optical density (OD) at 314 nm with an increasing concentration of TMS-β-CD or TMS-γ-CD macrocycles and the fitting according to a 1:1 host–guest complexation stoichiometry. Thus, the *K*_s_ in CHCl_3_ was determined to be approximately 205 and 150 (±30) M^−1^, respectively (see the determined *K*_s_ of **1b** in Figure 2).

Concerning the binding affinity of molecule **1** to the TMS-β-CD and TMS-γ-CD cavities, it should be mentioned that the values toward native γ-CD decrease [13]. Nevertheless, they are in good agreement with the reported stability constants of CDs with different molecules in organic solvents [36].

**4a** and **4b** polyrotaxanes and **4** non-rotaxane copolymer were obtained by firstly carrying out the well-known paladium-catalyzed Suzuki coupling reaction of acceptor moieties, which were either in the form of inclusion complexes into TMS-β-CD or TMS-γ-CD cavities (**1a**, **1b**) or non-complexed state **1** with **2** and **3** units in molar ratios 5:4:1 with toluene as a solvent. This was followed by the termination of the growing chains with Br–Ph to yield **4a** and **4b** polyrotaxanes and the **4** non-rotaxane copolymer. The encapsulation into permodified CDs cavities compared to those previously reported [13], i.e., the use of toluene as a solvent medium instead of a 1:1 v/v toluene/DMF mixture, led to copolymers soluble in toluene, THF, CH_2_Cl_2_ (DCM), and CHCl_3_. In addition, films characterized by a higher optical quality could be prepared by spin-coating from polyrotaxane solutions.

The chemical structure of the synthesized copolymers were proved by FTIR and NMR spectroscopy. The FTIR spectra of **4a** and **4b** (Figure S3 in Supporting Information File 1) show all the characteristic bands of **4** and additional bands located in the 1254–1045 cm^−1^ region, which correspond to TMS-β-CD and TMS-γ-CD. The peaks assigned to the C–H out-of-plane bending vibrations of the non-rotaxane **4** (814 cm^−1^) were at distinctly lower energy (750 and 748 cm^−1^) in **4a** and **4b** polyrotaxanes, respectively.

**Figure 3:**
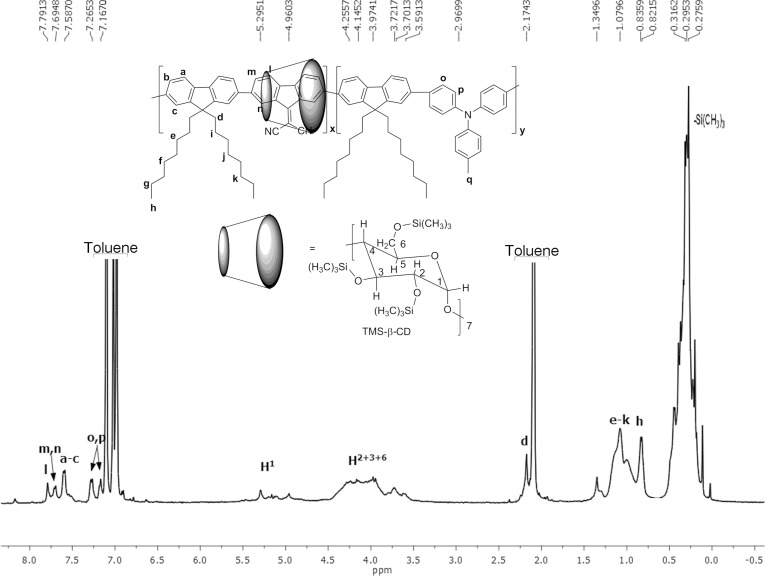
^1^H NMR spectrum of the polyrotaxane **4a** copolymer in toluene-*d*_8_.

Figure 3 displays the ^1^H NMR spectrum of the **4a** polyrotaxane copolymer with the assignments of the resonance peaks. The ^1^H NMR spectra of **4b** and non-rotaxane **4** are shown in Figures S4 and S5 in Supporting Information File 1.

The resonance peaks of l, m and n protons from monomer **1** are shifted upfield by more than 0.29 and 0.09 ppm in the polyrotaxane **4a** compared to those of the non-rotaxane **4** counterpart (Figure 3 and Figure S4 in the Supporting Information File 1). A comparison of the integrals of (l + m + n) protons from monomer **1** to those corresponding to H^1^ protons of TMS-β-CD and TMS-γ-CD facilitated the calculation of the average number of coverage per repeating unit. Based on the ratio of the integrated area of the H^1^ from TMS-β-CD (5.29 ppm, I*_H_^1^*) and the proton peaks of **1** (7.78–7.69 ppm, *I*_l+m+n_); (*I*_H_*^1^*/7)/(*I*_l+m+n_/6) the coverage ratio was found to be about 0.43 (ca. 43.2% coverage). A lower coverage ratio of ca. 37.6% was obtained for **4b** polyrotaxane, see Figure S5 in Supporting Information File 1. It should be pointed out that the lower coverage compared to native γ-CD [13] can be assigned to the poor hydrophobic–hydrophobic interactions of molecule **1** towards TMS-β-CD and TMS-γ-CD.

The physical properties of the copolymers are listed in Table 1.

**Table 1:** Molecular weight (*M*_n_ in g∙mol^−1^), polydispersity index *(M*_w_/*M*_n_), coverage ratio, and thermal properties of the copolymers.

Sample	*M*_n_^a^	*M*_w_/*M*_n_^b^	Coverage^c^	*T*_g_^d^ (°C)	*T*_d_^e^ (°C)

**4**	16400	1.58	–	91	414
**4a**	39870	2.16	0.43	128	412
**4b**	23195	2.34	0.37	105	413

^a^Number average molecular weight determined by GPC, THF, Pst standards. ^b^Polydispersity index. ^c^Average number of macrocycle molecules/structural units, determined from ^1^H NMR analysis. ^d^Glass-transition temperature estimated from the second-heating DSC measurements. ^e^Temperature resulting in a 5% weight loss based on the initial weight.

The molecular weights (*M*_n_) and molecular weight distributions (*M*_w_/*M*_n_) of copolymers were determined by gel permeation chromatography (GPC) analysis (not shown). **4a** and **4b** polyrotaxanes displayed a value of *M*_w_/*M*_n_ higher than the one of the **4** non-rotaxane sample, which hints at a different content of threaded TMS-β-CD or TMS-γ-CD on the copolymer chains (Table 1).

**Figure 4:**
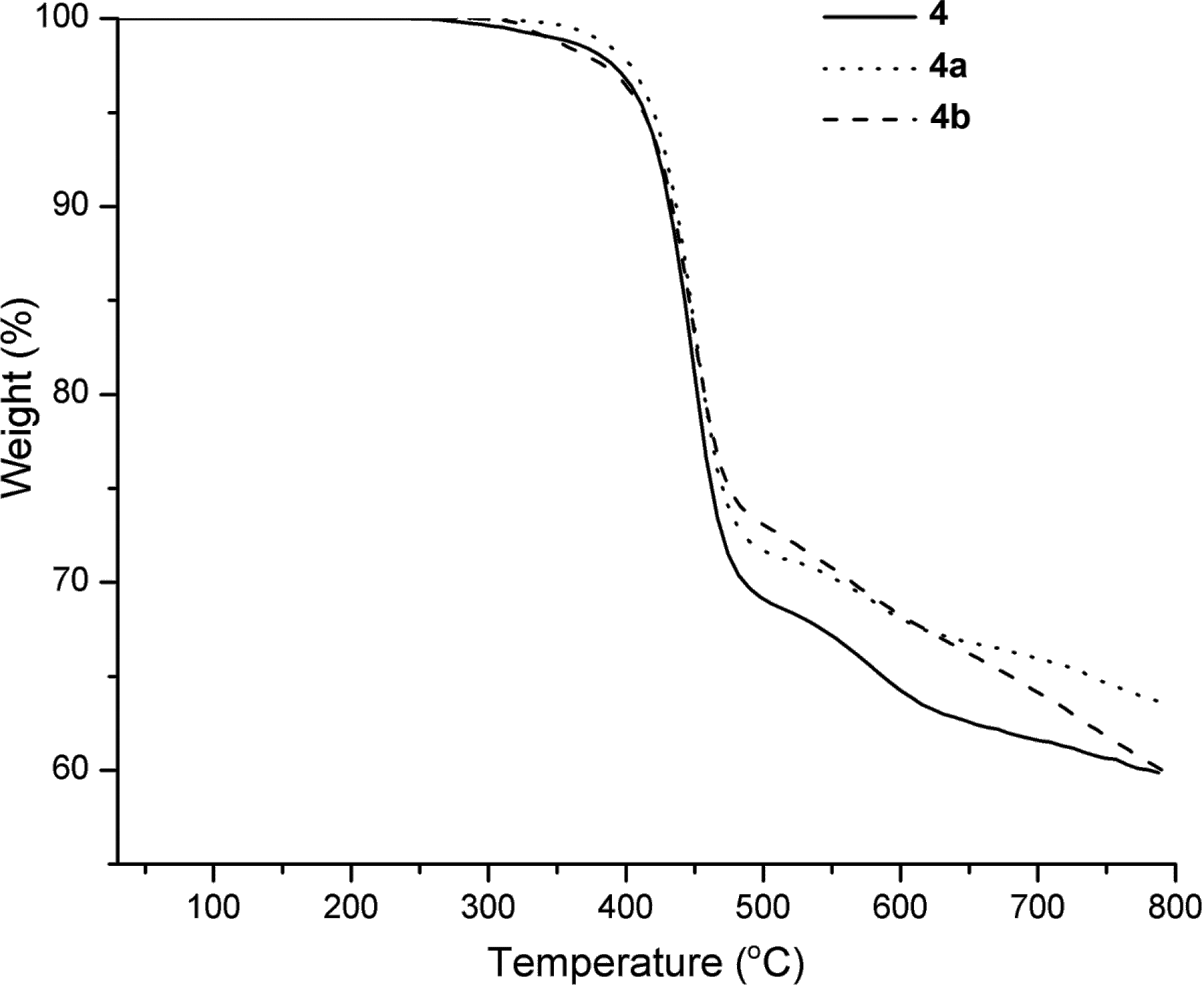
Termogravimetric curves (TG) of the copolymers.

The thermal properties of the copolymers were evaluated by thermogravimetric analysis (TGA) and differential scanning calorimetry (DSC). The results are summarized in Table 1. **4a** and **4b** polyrotaxanes exhibited a thermal decomposition temperature around 412 °C, whereas the thermal decomposition temperature of reference **4** was around 414 °C at 5% weight loss (Figure 4 and Table 1). It is noteworthy that the polyrotaxane formation increases the thermal stability of the macrocycle molecules. Moreover, the decomposition temperature at 5% weight loss for TMS-γ-CD determined by TGA analysis is around 390 °C [32].

**Figure 5:**
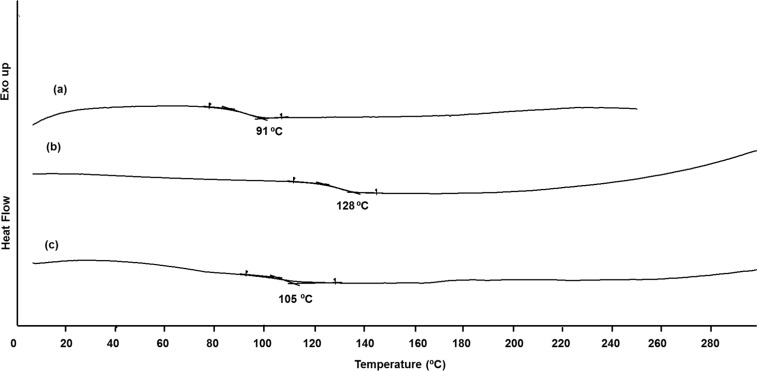
DSC traces on second heating scan of **4** (a), **4a** (b) and **4b** (c).

The DSC curves of the polyrotaxane samples showed glass-transitions temperature (Figure 5). The *T*_g_ values increase from 91 °C for **4** to 128 °C and 105 °C for **4a** and **4b**, respectively. The DSC results suggest that the encapsulation of molecule **1** into TMS-β-CD and TMS-γ-CD cavities leads to more rigid copolymer structures with an increased *T*_g_ (Table 1). It should be pointed out that the higher molecular weight of **4a** and **4b** copolymers can also influence the phase transition temperature.

**Figure 6:**
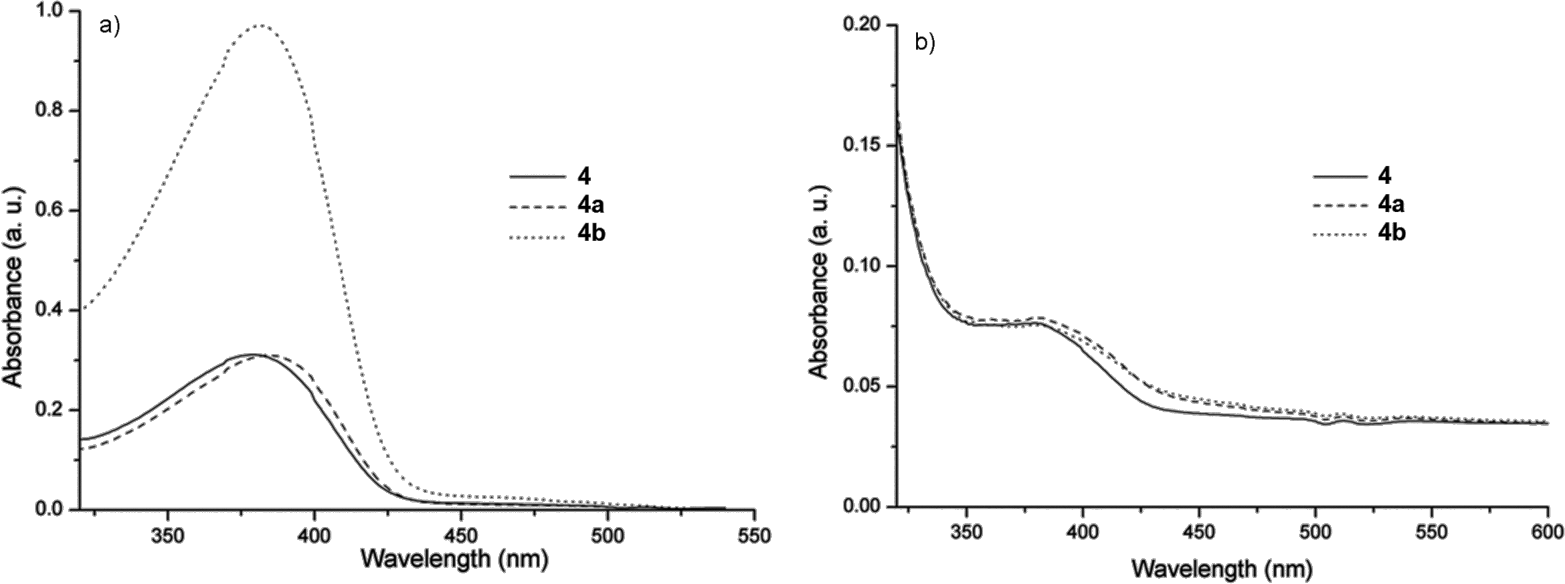
Optical absorption spectra of the copolymers in DCM solutions (*c* = 1.5∙10^−6^ mg∙mL^−1^) (a) and thin films (b).

**Figure 7:**
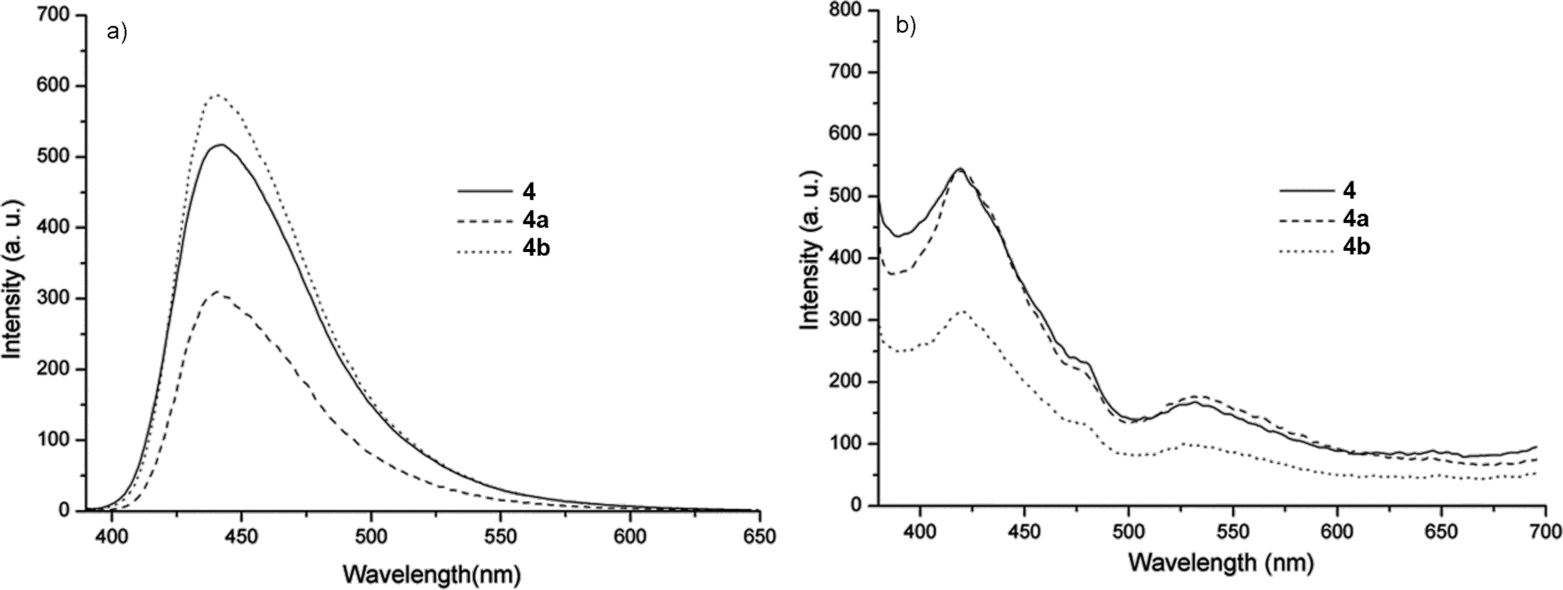
Fluorescence emission spectra of the copolymers in DCM solutions (*c* = 1.5∙10^−6^ mg∙mL^−1^) (a) and thin films (b).

The UV–vis absorption and fluorescence spectra of the copolymers **4**, **4a** and **4b** in DCM solutions and thin films are shown in Figure 6 and Figure 7, and the results are summarized in Table 2.

**Table 2:** Optical properties of copolymers.

Sample	λ_abs_^a^ (nm)	λ_em_^b^ (nm)	λ_abs_^c^ (nm)	λ_em_^d^ (nm)	Φ^e^ (%)	τ_1_^f^ (ns)	τ_2_^f^ (ns)	χ*^2^*^ g^	*E*_opt_^h^ (eV)

**4**	379	441	383	418; 531	7.00^i^	0.88	1.56	0.935	2.89
**4a**	383	443	385	418; 530	17.60	–	1.03	1.029	2.88
**4b**	381	442	383	419; 527	16.27	–	1.14	0.997	2.90

^a^Absorption from diluted DCM solutions [*c* = 1.5∙10^−3^ mg∙mL^−1^]. ^b^Emission from diluted DCM solutions [*c* = 1.5∙10^−3^ mg∙mL^−1^]. ^c^Absorption of films spin-coated from DCM solutions. ^d^Emission of films spin-coated from DCM solutions. ^e^Independently measured PL quantum efficiency in diluted DCM solutions [*c* = 1.5∙10^−3^ mg∙mL^-1^]. ^f^Lifetimes. ^g^Chi square. ^h^The optical gap, *E*_opt_, estimated from the onset of absorption (*E*_opt_ = 1240/λ_onset)_*_. _*^i^Data taken from [13].

Figure 6 shows the UV–vis absorption spectra of the copolymers in dilute DCM solution (a) and thin films (b). The absorption bands of the copolymers are associated with π–π^*^ transitions and can be correlated to the degree of polymer ordering [37]. Polyrotaxane copolymers exhibited absorption bands with a slight red shift of 4 nm for **4a** and 2 nm for **4b** with respect to the non-rotaxane **4** counterpart (Figure 6 and Table 2). This suggests that the intramolecular charge transfer between **1** and **2** units of 1/4 molar ratios is relatively weak. Moreover, the smaller red shift from a dilute solution compared to the solid-state of **4a** and **4b** polyrotaxanes corroborated the beneficial effect of TMS-β-CD and TMS-γ-CD encapsulations on the lower aggregation tendency [13].

The determined absorption onset wavelength of **4**, **4a** and **4b** copolymer films are 429, 430 and 427 nm, which gives rise to the corresponding optical band gaps (*E*_opt_ = 1240/λ*_onset_*) [38], of 2.89, 2.88 and 2.90 eV, respectively Table 2. The fluorescence emission (PL) of the copolymers **4**, **4a** and **4b** in dilute DCM solutions show emission maxima at 441, 443 and 442 nm (Figure 7a), while it is around 418 nm in the solid state with a shoulder at around 530 (Figure 7b and Table 2). The emission at shorter wavelengths can be attributed to the conjugated PF backbones, whereas the band at longer wavelengths is caused by weakly coupled aggregates or the formation of excimers [39].

**Figure 8:**
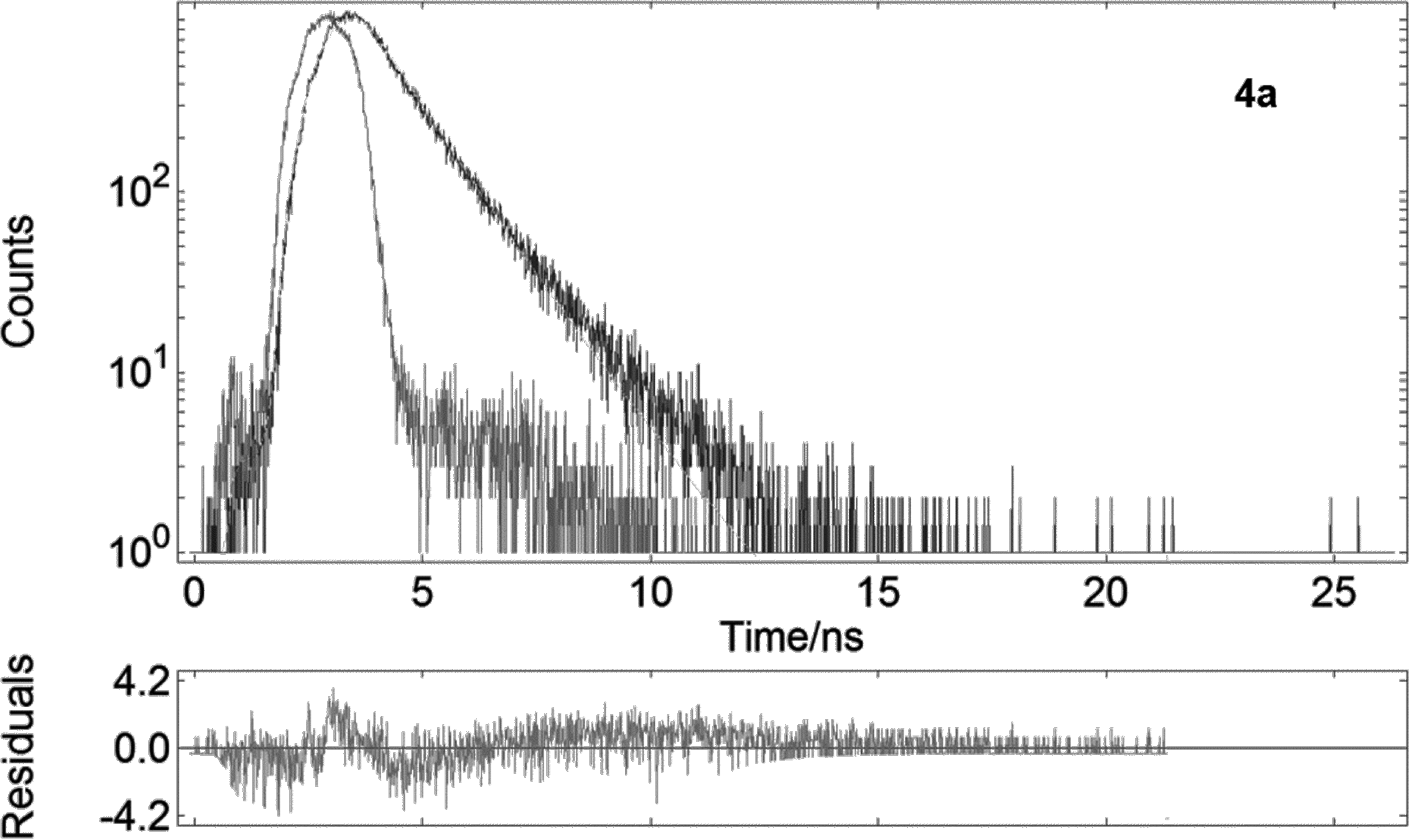
Fluorescence lifetime decay traces of **4a** polyrotaxane at 440 nm in DCM solution.

To gain further insight into the effect of macrocyclic encapsulation the fluorescence quantum (Φ) yield was estimated with an integrating sphere at an excitation wavelength of 380 nm (Table 2). The DCM solution of **4a** and **4b** copolymers suggested no improvements of Φ compared with previously reported results [13], which restrict us to point out a distinctive effect of the rotaxane formation with TMS-β-CD and TMS-γ-CD macrocycles. To further understand Φ results, we also carried out fluorescence intensity decay. The decay traces of **4a** and **4b** showed a single exponential kinetics with τ = 1.03 and 1.14 ns (Table 2, Figure 8 and Figure S6 in Supporting Information File 1). A single exponential kinetics has also been observed for other encapsulated systems and can be attributed to a relatively strong interaction between macrocyclic molecules and conjugated cores [26,40]. The decay traces of **4** non-rotaxane counterpart obtained from the fluorescence lifetime measurements follow a bi-exponential decay, which consists of a main component with a relative short time of τ_1_ = 0.88 (57.08%) and a minor component with a longer lifetime of τ_2_ = 1.56 ns (42.92%) (Figure S7 in Supporting Information File 1 and Table 2). The observed behavior suggests that the bi-exponential decay of the non-rotaxane **4** may be caused by the intrachain emission and excitonic lifetime, whereas in the case of the polyrotaxanes it may only be attributed to the excitonic contribution [39].

**Figure 9:**
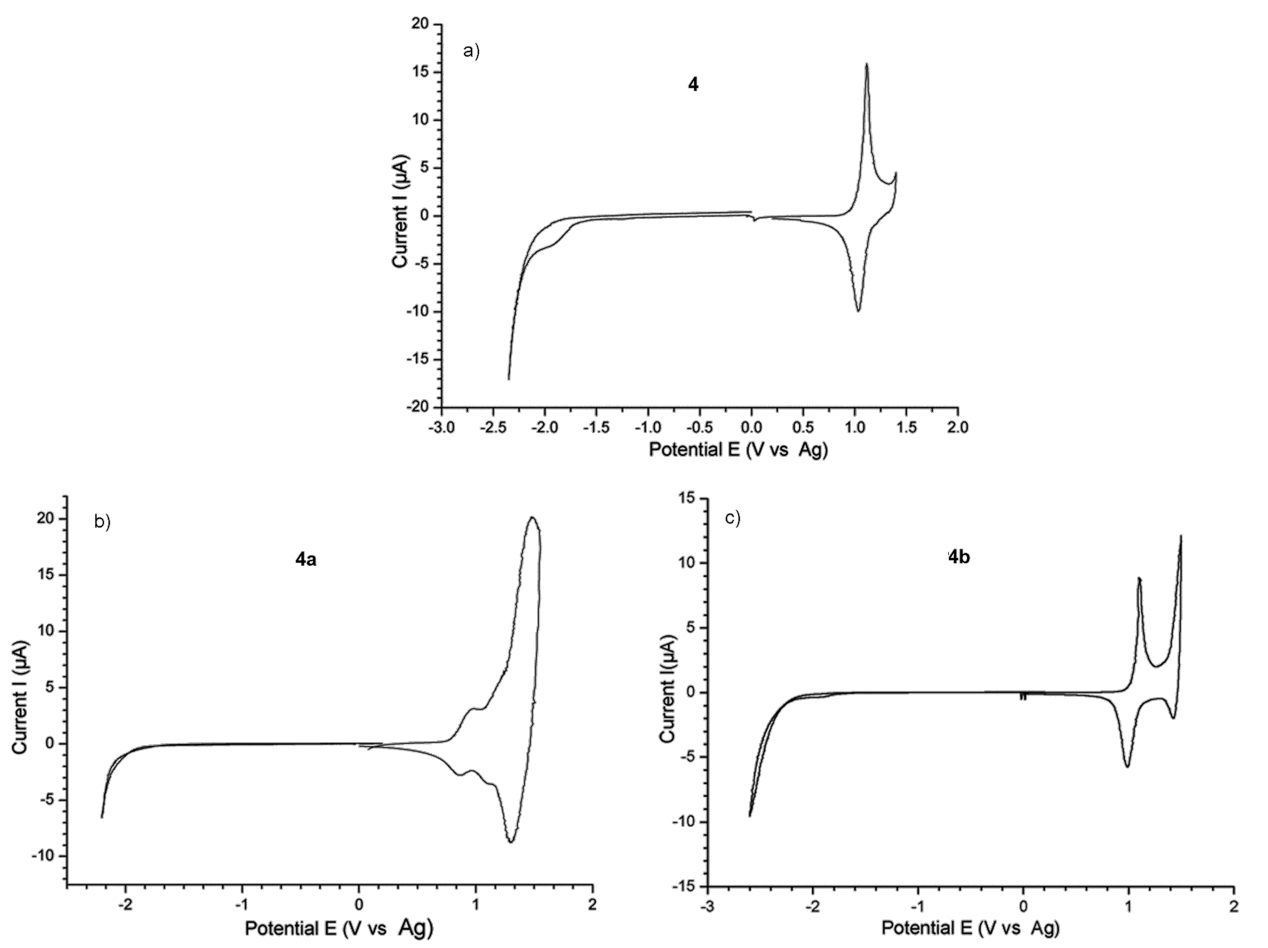
CV of **4** (a), **4a** (b) and **4b** (c) in 0.1 M TBAP/ACN solution at scan rate 20 mV∙s^−1^ copolymer films.

To further understand the electronic properties of the copolymers, the redox properties, i.e., the oxidation *E*_ox_ and the reduction potentials *E*_red_ of the copolymers were investigated by cyclic voltammetry (CV) (Figure 9 and Table 3). CV experiments have been widely employed to investigate the solid state redox behavior of some hole-transporting layers [41], semiconducting polymer layers [13,29–30,42], or electrodeposited layers [43]. In order to remove any precedent electrochemical processes, which could change the film morphology by the insertion of counter ions and solvent molecules, both *n*- and *p*-doping processes were performed independently with fresh copolymer films.

**Table 3:** The redox properties of **4**, **4a** and **4b** copolymers.

Sample	**4**	**4a**	**4b**

Oxydation^a^ (*E*_ox___onset_) (V)	1.04	0.82; 1.15	1.05
Reduction^b^ (*E*_red___onset_) (V)	−1.75 −2.10	−2.02	−2.09
IP ~ *E*_HOMO_^c^ (eV)	−5.4	−5.51	−5.41
EA ~ *E*_LUMO_^d^ (eV)	−2.26	−2.43	−2.27
Δ*E*_g_^e^ (eV)	3.14	3.08	3.13

^a^Onset oxidation potentials. ^b^Onset reduction potentials. ^c^Ionization potential determined according to the equation: IP = +e (*E*_p,onset_ − *E_a_*^1/2^_Fc_) + IP_Fc_, where *E*_a_^1/2^_Fc_ is the half-potential for the reversible reduction reaction of the redox couple (Fc^+^/Fc) and the value of the ionization potential of Fc (IP*_Fc_*) = 4.76 eV [44]. ^d^Electron affinity determined according to the equation: EA = +e (*E*_n,onset_ – *E*_c_^1/2^_Fc_) + EA_Fc_*^+^*, where *E*_c_^1/2^_Fc_ is the half-potential for the reversible oxidation reaction of the redox couple (Fc^+^/Fc), and the value of the electronic affinity of Fc cation (EA_Fc_*^+^*) = 4.76 eV [44]. ^e^Electrochemical gap (Δ*E*_g_) = EA − IP.

The CV of **4**, **4a** and **4b** samples exhibited oxidation and reduction processes (Figure 9). These measurements allow us to estimate the ionization potential (IP), the electronic affinity (EA), and the electrochemical band gaps (Δ*E*_g_) by using ferrocene (Fc) as a reference [45–46]. It should be mentioned that the encapsulated **1a** or **1b** moieties and **2** randomly distributed into the **3** backbone leads to the synthesis of compounds with smaller optical and electrochemical gaps, IP and EA compared to any of the constituents, which is necessary for electronic applications.

The diagram with HOMO/LUMO levels and the work function of the indium tin oxide (ITO) coated glass substrates with poly(3,4-ethylenedioxythiophene):poly(styrenesulfonate) (PEDOT:PSS) (anode) and Ca or Al (cathode) indicates that the compounds may be suitable for the hole and electron transport (HTL) into the PLED active layer [47] (Figure S8 in Supporting Information File 1).

**Figure 10:**
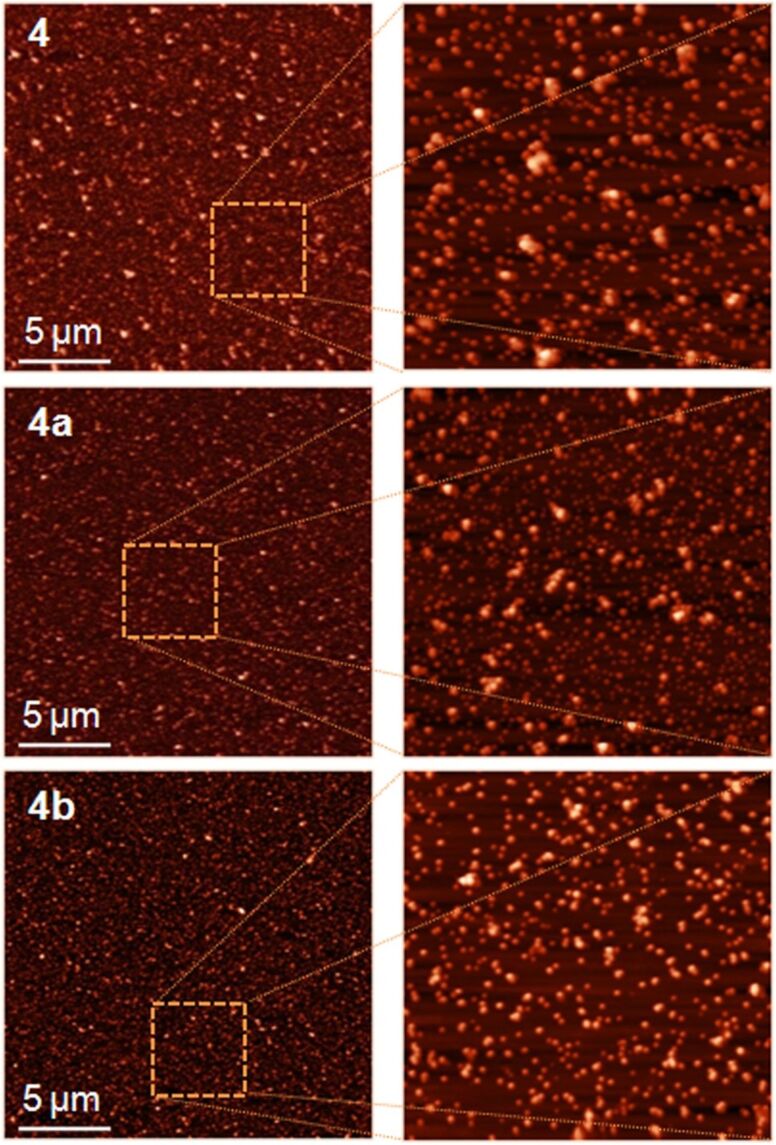
Representative AFM images obtained over 20 × 20 and 5 × 5 µm^2^ areas of the non-rotaxane **4**, **4a** and **4b** polyrotaxanes.

**Figure 11:**
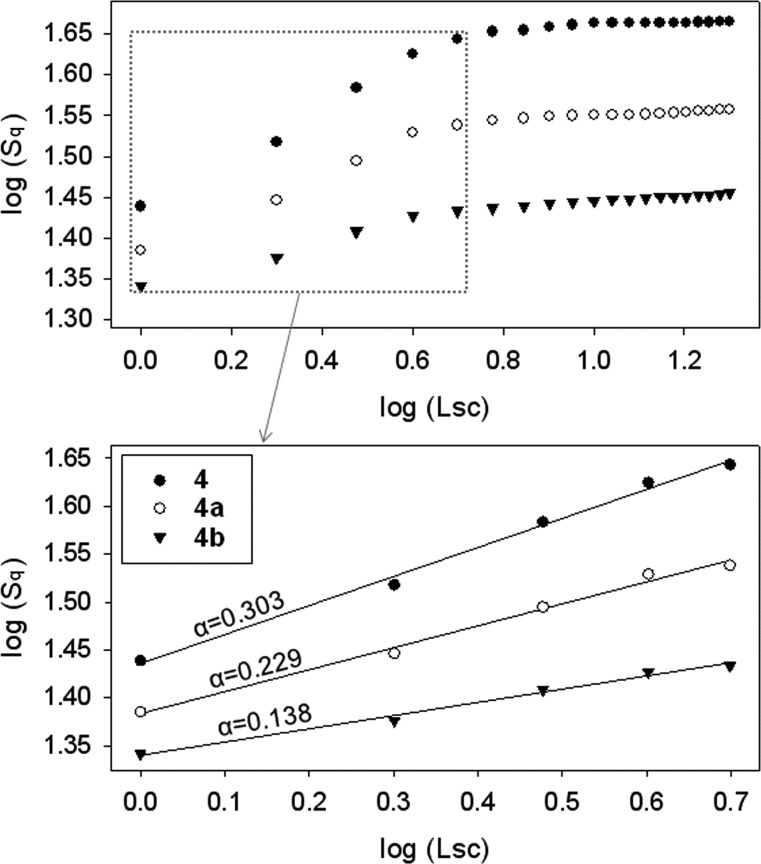
The roughness exponent α calculated as the slope of log(*S*_q_) versus log(Lsc) for the reference **4**, **4a** and **4b** polyrotaxanes.

To gain further insight into the beneficial effect of TMS-β-CD and TMS-γ-CD encapsulations, the surface topography was also investigated by atomic force microscopy (AFM) analysis. Multiple scans with sides of 1 to 20 μm were carried out over square areas. Selected representative images obtained for the non-rotaxane **4**, **4a** and **4b** polyrotaxanes over areas of 20 × 20 µm^2^ and 5 × 5 µm^2^ are shown in Figure 10. Based on the calculated root mean square roughness (*S*_q_) for all explored areas we derived the roughness exponent (α), employed for an accurate comparison of the surface characteristics, from the slope of the log(*S*_q_) versus the scan length in double logarithmic plot curves, log(Lsc), before saturation (Figure 11) [48]. For a large scan length, each curve turns into a plateau. The non-rotaxane **4** film surface displays granular morphology with grain diameters of 99 ± 17 nm. The dispersity of grain sizes was supported by a higher roughness exponent of 0.303 (Figure 11). **4a** and **4b** polyrotaxanes also show a granular morphology and their values of the α parameter and the grain diameter reflect the chemical changes of the conjugated polymer surfaces. Thus, the **4a** film surface shows smaller grains with an average diameter of 86 ± 8 nm. Furthermore, the relative uniform grain size dispersion induces the lower value of α (0.229), which also provides microscopic evidence of the changes on the morphological characteristics.

On the other hand, the sample **4b** shows larger grain formations with an average diameter of 95 ± 5 nm, which is attributed to the lower content of **1b** encapsulated molecules. These grains have a uniform size dispersity and distribution, which results in a rather small value of α (α = 0.138). Taking into account all the information obtained from the topographical investigations, it can be concluded that the formation of the rotaxane architecture generally improves the morphological characteristics of the film surfaces.

## Conclusion

The synthesis and photophysical properties of two conjugated polyrotaxanes containing electron-accepting units encapsulated into TMS-β-CD or TMS-γ-CD cavities and electron-donating moieties, statistically distributed into the conjugated chains of 9,9-dioctylfluorene were investigated and compared to those of the corresponding non-rotaxane counterpart. The encapsulation into TMS-β-CD or TMS-γ-CD cavities leads to distinct improvements in the solubility in common organic solvents compared with native γ-CD, molecular weights, increased glass-transition temperatures, enhancements of the transparency of the solid films, as well as the fluorescence efficiency and surface characteristics compared to those of the non-rotaxane counterpart. HUMO/LUMO energy levels proved that all copolymers are electrochemically accessible as electron-transporting materials. Our attempts to explore these compounds for polymer light-emitting layers and photovoltaic applications are currently underway and will be reported in due course.

## Experimental

### Materials and methods

9,9-dioctylfluorene-2,7-bis(trimethyleneborate) (**3**), 2,7-dibromofluoren-9-one, tetrakis(triphenylphosphine) palladium(0) [Pd(PPh_3_)_4_], β-CD and γ-CD, bromobenzene (Br–Ph), dimethylformamide (DMF), and dimethyl sulfoxide (DMSO) were purchased from (Sigma-Aldrich) and used as received. Malononitrile (Merck), tetrabutylammonium perchlorate (TBAP) for electrochemical analysis (99.0%) (Fluka) were used without further purification. Acetonitrile (ACN) (Fischer), DCM, CHCl_3_, toluene and all other solvents were purchased from commercial sources (Sigma-Aldrich, Fisher) and used without further purification.

^1^H NMR spectra were recorded in toluene on a Bruker Advance 400 MHz instrument. The FTIR (KBr pellets) spectra were obtained on a Bruker Vertex 70 spectrophotometer. The molecular weights of copolymers were determined by GPC in THF by using a Water Associates 440 instrument and polystyrene (Pst) calibrating standards. TGA analysis was performed under a constant flow of nitrogen (20 mL·min^−1^) with a heating rate of 10 °C·min^−1^ and using a Mettler Toledo TGA/SDTA 851e balance. The heating scans were performed on 1.5 to 3 mg of the sample in the temperature range from 25 to 800 °C. DSC was performed with a Mettler Toledo DSC-12E calorimeter with two repeated heating-cooling cycles at a heating rate of 10 °C·min^−1^ under N_2_ atmosphere. UV–vis absorption spectra were recorded on a SPECORD 200 Analytik Jena spectrometer with 10 mm quartz cells. Fluorescence measurements were carried out an on Perkin Elmer LS 55 spectrometer. The excitation wavelength corresponds to the maximum absorption band. Time-resolved fluorescence data were acquired with an Edinburgh FLS 980 photoluminescence spectrometer with 1 cm quartz cells. A 375 nm pulsed diode laser (EPL-375, maximum average power: 5 mW, pulse width: 73.2 ps) was used as a light source. Decay data analysis was performed by the deconvolution procedure with multiexponential decay models. The quality of the fit was estimated by the parameter χ*^2^* (0.90 ≤ χ*^2^* ≤ 1.10) and the symmetrical distribution of the residuals about the zero axis. All measurements were performed at room temperature.

The fluorescence quantum yield was determined by using the FLS 980 fluorospectrometer with an integrating sphere and with 380 nm excitation wavelength.

Cyclic voltammograms (CV) were carried out in a three-electrode cell in which Pt (1 mm diameter) was used as a working electrode, a Pt-wire as counter-electrode and a Ag wire as a pseudo-reference electrode. 0.1 M TBAP solution in anhydrous ACN was used as the supporting electrolyte. The set-up was introduced into a glove box and controlled by AUTOLAB PGSTAT 101 (Ecochemie) by using NOVA software. The pseudo-reference was calibrated with a 10^−3^ M of Fc solution in ACN. The polymer samples were drop-casted onto the working electrode from a concentrated DCM solution and studied in the interval −2.5 and +2.0 V vs Ag wire. Cathodic and anodic scans were performed independently.

Atomic force microscopy (AFM) images were collected in semi-contact mode with a Solver PRO-M (NT-MDT Co, Zelenograd, Moscow, Russia) by means of a commercially available NSG03 rectangular-shaped silicon cantilever with a resonance peak of 88 kHz. For image acquisition from different areas (squares with scanning length, Lsc, ranging from 1 μm to 20 μm), the Nova v.1.26.0.1443 for Solver software was used. The root mean square roughness (*S*_q_) was calculated for all the investigated areas. The roughness exponent, α, was calculated as the slope of roughness *versus* scan length in a double logarithmic plot, log(Lsc), before saturation.

**Synthesis of TMS-β-CD and TMS-γ-CD:** TMS-β-CD and TMS-γ-CD were obtained by the silylation of native β-CD and γ-CD with 1-trimethylsilylimidazole [32].

**Synthesis of 2,7-dibromo-9,9-(dicyanomethylene)fluorene (1):** 2,7-dibromo-9,9-(dicyanomethylene)fluorene was prepared by a condensation reaction between 2,7-dibromofluoren-9-one with malononitrile at 110 °C in DMSO according to a literature method [13,33–34].

**Synthesis of bis(4-bromophenyl)(4-methylphenyl)amine (2):** Bis(4-bromophenyl)(4-methylphenyl)amine was synthesized according to a previously described procedure [13,35].

**Synthesis of 4a polyrotaxane copolymer:** In the round bottomed flask equipped with a reflux condenser and a Dean–Stark trap, 1.061 g (0.4 mmol) of TMS-β-CD dissolved in toluene (6 mL) and 0.0773 g (0.2 mmol) of monomer **1** were added and stirred at room temperature in the dark under nitrogen atmosphere for 72 h to give a dispersion. Then 0.334 g (0.8 mmol) of **2**, 0.577 (1 mmol) of **2**, 2 mL of 3 M Na_2_CO_3_ and 20.8 mg (1.82 × 10^−2^ mmol) of (Ph_3_P)_4_Pd(0), dissolved in 5 mL of degassed toluene, were added. The flask was equipped with a condenser; evacuated and filled with nitrogen several times to remove traces of air and the mixture was vigorously stirred in the dark under nitrogen atmosphere for 72 h at 95–100 °C. An excess of 0.0287 g (0.05 mmol) of monomer **3** dissolved in 3 mL of toluene was then added, and the reaction was continued for 10 h in order to obtain the macromolecular chains terminated with borate units. Finally, 1.0 μL of Br–Ph was added as end-capper of the copolymer chain, and the reaction was continued overnight. After cooling, the mixture was extracted with DCM. The organic extracts were washed with water and dried over magnesium sulfate. The DCM solution was concentrated by rotary evaporation and precipitated in hexane to remove free TMS-β-CD. The yellow polymer sample was filtered. After drying the solid was purified by Soxhlet extraction with methanol for 16 hours, dissolved in toluene and precipitated with methanol, filtered, washed with acetone, and dried. 0.301 g (35.5% yields) of a yellow solid was obtained after drying. FTIR (KBr, cm^−1^): 3439, 2958, 2853, 2370 (CN), 2344, 1721, 1632, 1412, 1367, 1319, 1251, 1147, 1096, 1045, 966, 885, 841, 750, 686, 580; ^1^H NMR (toluene-*d*_8_) 7.79 (s, 2H), 7.69 (d, 4H), 7.58 (s, 6H), 7.26–7.16 (d, 12H), 5.29–4.96 (m, 7H^1^, TMS-β-CD), 4.25–3.59 (m, 28H, H*^2+3+6^*, TMS-β-CD), 2.17 (s, 4H), 1.07 (m, 48H), 0.83 (s, 12H), 0.31–0.27 (s, 189H, TMS-β-CD).

**Synthesis of 4b polyrotaxane copolymer: 4b** was synthesized by similar experimental conditions as described above except that TMS-γ-CD was used instead of TMS-β-CD. The copolymer was obtained as a yellow solid in 31.3% yield. FTIR (KBr, cm^−1^) 3439, 2957, 2925, 2854, 2371 (CN), 2343, 1743, 1611, 1541, 1510, 1462, 1440, 1413, 1366, 1298, 1251, 1152, 1097, 1045, 966; ^1^H NMR (toluene-*d*_8_) 0.78 (s, 2H), 7.71–7.69 (s, 4H), 7.58 (s, 6H), 7.29–7.17 (d, 12H), 5.29–4.96 (m, 8H^1^, TMS-γ-CD), 4.23–3.72 (m, 32H, H*^2+3+6^*, TMS-γ-CD), 2.17 (s, 4H), 1.07–0.92 (m, 48H), 0.83 (s, 12H), 0.27 (s, 216 H, TMS-γ-CD).

**Synthesis of the non-rotaxane 4 copolymer counterpart:** The copolymer **4** was synthesized according to the previously reported procedure except that toluene was used as reaction solvent instead of a mixture of 1:1 v/v toluene/DMF [13]. FTIR (KBr, cm^−1^): 3432, 3028, 2924, 2852, 2372 (CN), 2345, 2222, 1719, 1599, 1509, 1462, 1408, 1375, 1319, 1282, 1180, 1014, 889, 814, 752, 721, 582, 516; ^1^H NMR (toluene-*d*_8_) 7.82 (s, 2H), 7.73 (d, 4H), 7.62–7, 57 (s, 6H), 7.32–7.20 (d, 12H), 2.21 (s, 4H), 2.16 (s, 3H), 1.11 (s, 48H), 0.87 (s, 12H).

## Supporting Information

FTIR spectra of the TMS-β-CD and copolymers, ^1^H NMR spectra of the TMS-β-CD, non-rotaxane **4** and **4b** polyrotaxane copolymers, the fluorescence lifetimes of the non-rotaxane **4** and **4b** polyrotaxane copolymers, the diagram with HOMO/LUMO levels of the compounds in addition to the work function of the indium tin oxide (ITO) coated glass substrates with poly(3,4-ethylenedioxythiophene):poly(styrenesulfonate) (PEDOT:PSS) (anode) and Ca or Al (cathode) are available.

File 1 Characterization data of the compounds: FTIR, ^1^H NMR, fluorescence lifetimes and the diagram with HOMO/LUMO energy levels of the copolymers.
