# Supplementary material for: The effect of permodified cyclodextrins encapsulation on the photophysical properties of a polyfluorene with randomly distributed electron-donor and rotaxane electron-acceptor units
Source: Beilstein J Org Chem. 2014 Sep 9;10:2145–56. doi: 10.3762/bjoc.10.222 (PMC4168896; doi:10.3762/bjoc.10.222)
Supplement: File 1 — Characterization data of the compounds: FTIR, 1H NMR, fluorescence lifetimes and the diagram with HOMO/LUMO energy levels of the copolymers. [file Beilstein_J_Org_Chem-10-2145-s001.pdf]

Supporting Information

for

The effect of permodified cyclodextrins encapsulation on the photophysical properties of a polyfluorene with randomly distributed electron-donor and rotaxane electron-acceptor units

Aurica Farcas[1]*, Ana-Maria Resmerita[1], Pierre-Henri Aubert[2], Flavian Farcas[3], Iuliana Stoica[1] and Anton Airinei[1]

Address: [1] Inorganic Polymers, ''Petru Poni'' Institute of Macromolecular Chemistry, Grigore Ghica Voda Alley, 700487 Iasi, Romania, [2] Laboratoire de Physicochimie des Polymères et des Interfaces (EA 2528), Institut des Matériaux, Université de Cergy-Pontoise, F-95031 Cergy-Pontoise Cedex, France and [3]"Gh. Asachi" Technical University, 61–63 Mangeron Blvd, 700050 Iasi, Romania

Email: Aurica Farcas - afarcas@icmpp.ro

* Corresponding author

Characterization data of the compounds: FTIR, ^{1}H NMR, fluorescence lifetimes and the diagram with HOMO/LUMO energy levels of the copolymers

Table of Contents

FTIR spectrum of TMS-β-CD macrocyclic molecule S2

^{1}H NMR spectrum of TMS-β-CD macrocyclic molecule S4

FTIR spectra of the reference **4**, **4a** and **4b** polyrotaxane copolymers S5

^{1}H NMR spectrum of the non-rotaxane **4** copolymer S6

^{1}H NMR spectrum of the polyrotaxane **4b** copolymer S7

The fluorescence lifetime of the non-rotaxane **4** copolymer S8

The fluorescence lifetime of the polyrotaxane **4b** copolymer S8

The diagram with HOMO/LUMO levels S9

References S10

S1. FTIR spectrum of TMS-β-CD macrocyclic molecule

The total silylation of native β-CD and γ-CD was proved using FTIR and H NMR spectroscopy of the resulting TMS-β-CD and TMS-γ-CD macrocyclic compounds, see Figures S1 and S2.

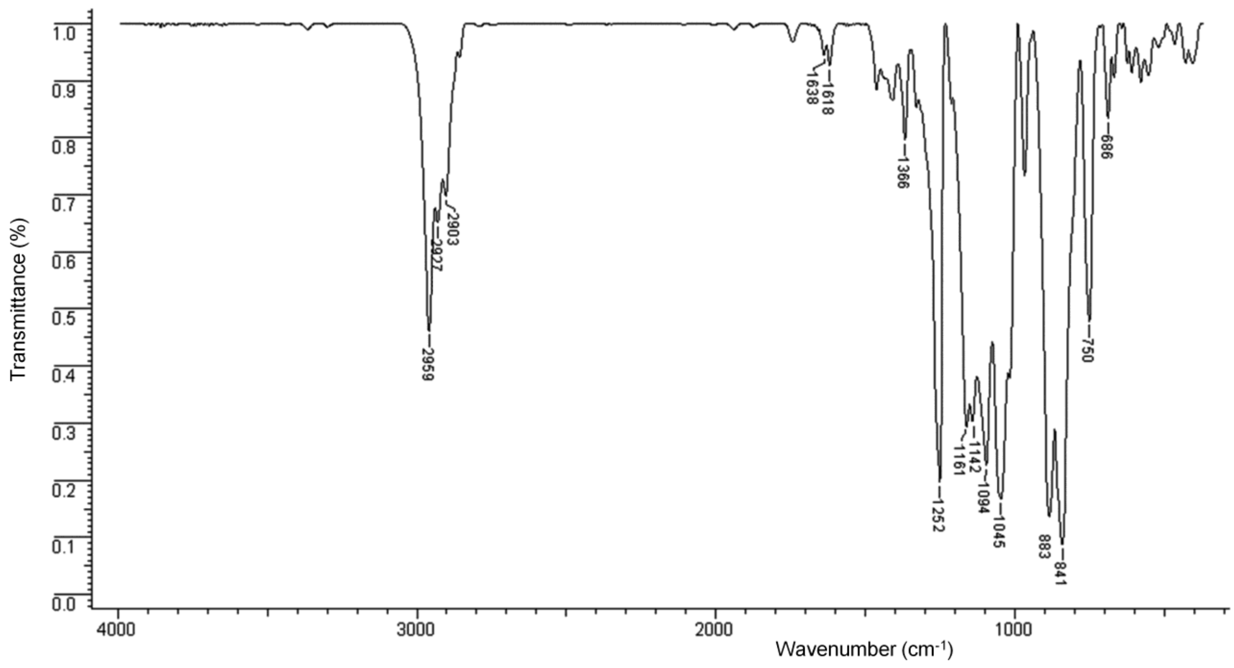

Figure S1: FTIR spectra of TMS-β-CD macrocyclic molecule.

FTIR spectra of both macrocycles exhibited characteristic bending band at 841cm^{-1} , attributed to C^1 and one split into two stretching bands at 1142-1161 cm^{-1} (C^1– O). C-O-C and Si-O-C give superposed bands at 1049-1096 cm^{-1} interval, while Si-CH_3 bonds presents absorption at 1252 cm^{-1}, see Figure S1. Both FTIR spectra of macrocycles did not show a band around 3500 cm^{-1}, characteristic to OH groups, see FTIR spectrum of TMS-β-CD in Figure S1.

S2. ^{1}H NMR spectrum of the TMS-β-CD

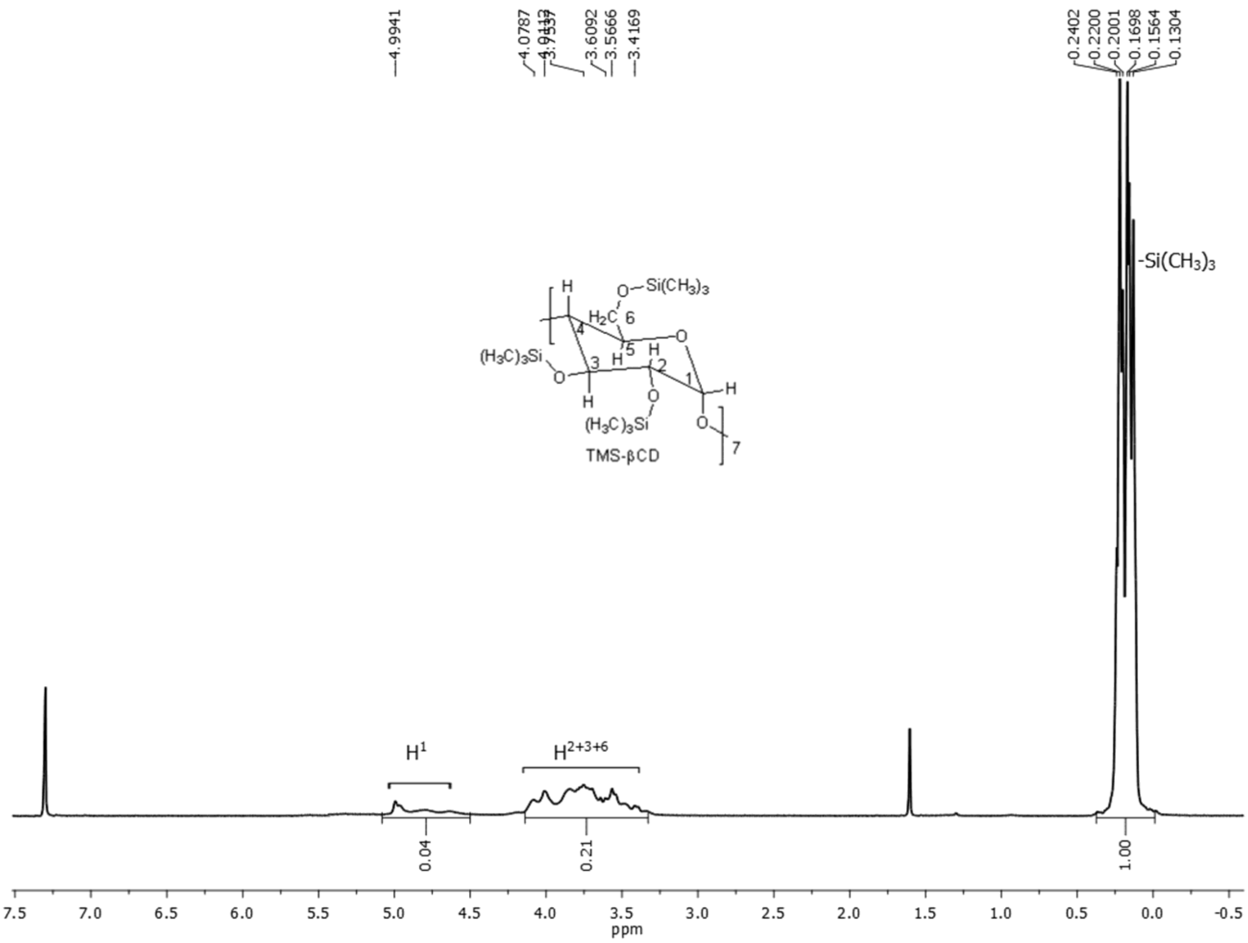

Figure S2: ^{1}H NMR spectrum of the TMS-β-CD with assignments of the resonance peaks in $CDCl_3$.

^{1}H NMR spectra of TMS-β-CD as well as TMS-γ-CD (not shown) indicated that the characteristic signals of OH protons from native CDs disappeared and a multitude of singlet peaks corresponding to trimethylsilyl units appeared around 0.08-0.18 ppm. The H^1 anomeric proton appeared as a doublet peak at 4.95 ppm in TMS-β-CD, see ^{1}H NMR spectrum of TMS-βCD in Figure S2.

S3. FTIR spectra of the reference **4**, **4a** and **4b** polyrotaxane copolymers

Figure S3 compares the FTIR spectra of the non-rotaxane **4**, and polyrotaxanes **4a** and **4b**. FTIR spectra of all polymers exhibited characteristics stretching vibration at about 2924 cm^{-1} (aromatic β C-H and ϕ C-H stretching), 2852 cm^{-1} (aliphatic C-H stretching) that are shifted towards lower frequencies in the spectra of **4a** and **4b** encapsulated compounds. In addition, cyan groups in **4** shows a short band at 2345 cm^{-1} that is shifted at 2344 and 2343 cm^{-1} in the spectra of **4a** and **4b** polyrotaxanes. Some strong (1719, 1599, 1509, 1462 cm^{-1}) bands of **4** are slightly shifted (2-4 cm^{-1}) to the lower frequencies in **4a** and **4b** polyrotaxanes. Surprisingly, in all FTIR spectra can be observed a band at approximately 3432 cm^{-1}. Therefore, its presence in the FTIR spectra of **4a** and **4b** polyrotaxanes (not present in the FTIR spectra of TMS-β-CD and TMS-γ-CD macrocycles), can not be attributed to the partial removal of the trimethylsilyl groups during synthesis or purification. The authors would rather suggest the presence of a small amount of water that was not properly removed during the drying of polymer samples, which can be responsible for the presence of this band in the FTIR spectra of all copolymers.

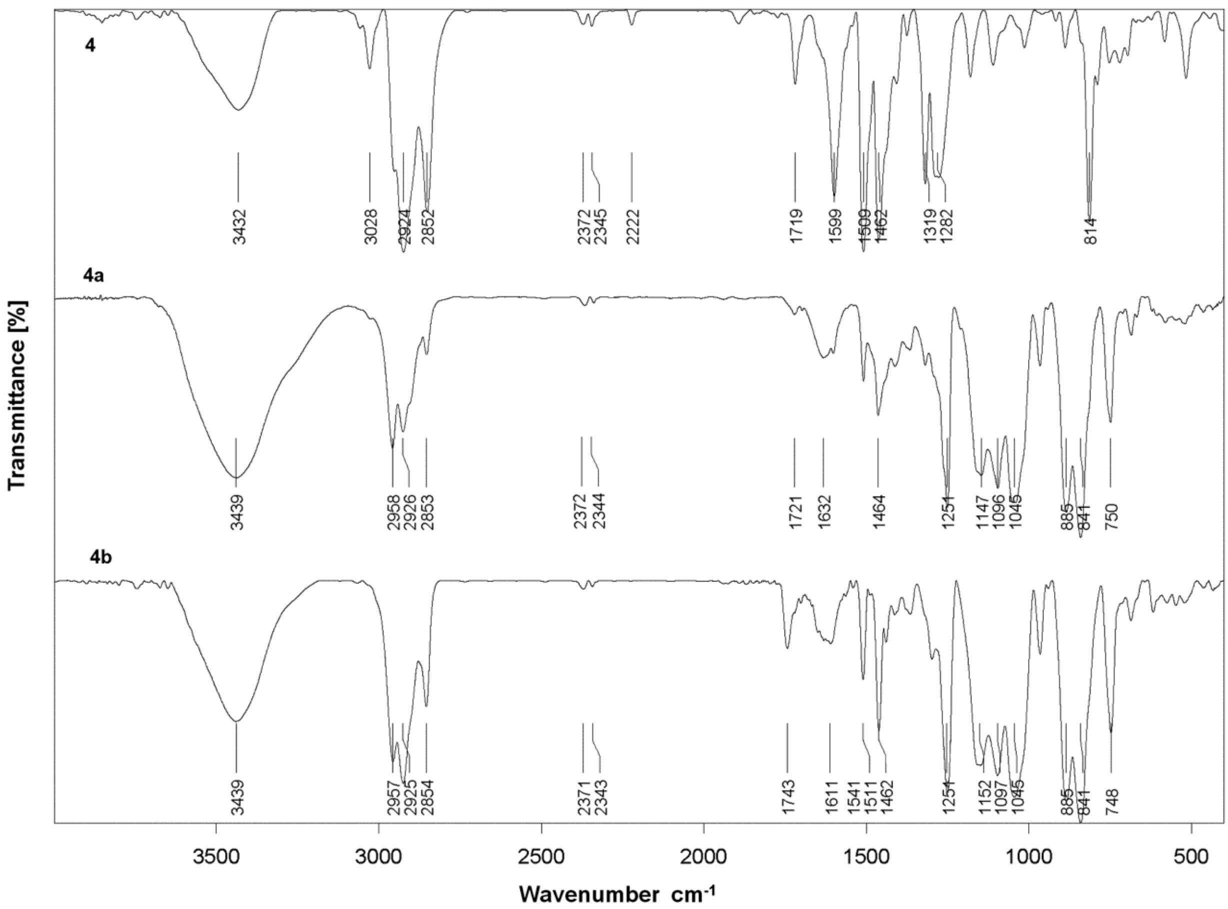

Figure S3: FTIR spectra of the reference **4**, **4a** and **4b** polyrotaxane copolymers.

S4. ^{1}H NMR spectrum of the non-rotaxane **4** copolymer

The ^{1}H NMR spectrum of the non-rotaxane **4** is presented in Figure S4, where resonance peak assignments are indicated. The ^{1}H NMR spectra of the non-rotaxane **4** was in good agreement with the proposed structures. The incorporation ratio was checked by comparing the relative ^{1}H NMR signal intensities of the protons resonances from **1** (δ = 7.83-7.73 ppm) with the protons signals of **2** (δ = 7.30-7.32 ppm). The results show an increase oxidative coupling reaction rate of **1** compared to **2**, a normal result owing to the negative mesomeric effect ($-M_S$) of dicyano groups from **1**. Thus, feed

composition of **1**/**2** was 1/4, while from ^{1}H analysis it was found 14.4/35.6, in the same range as previously reported results [1].

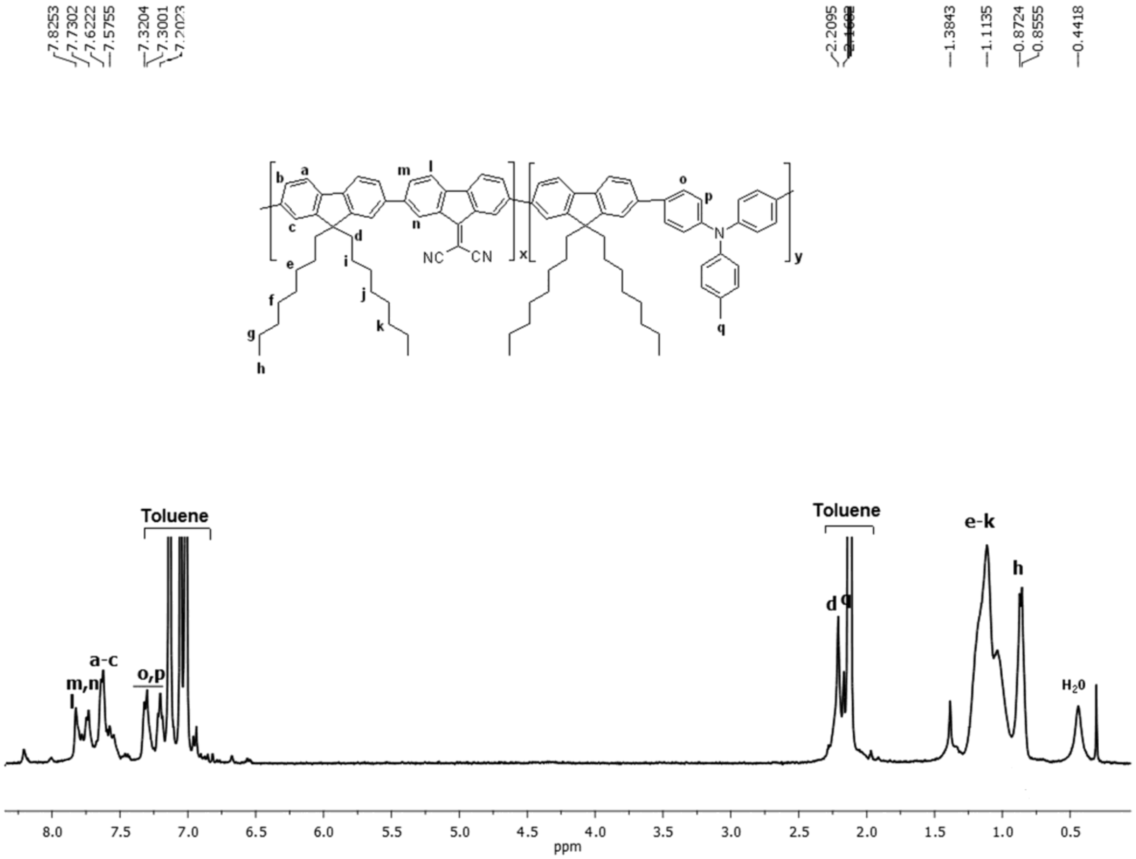

Figure S4: ^{1}H NMR spectrum of the non-rotaxane **4** copolymer with assignments of the resonance peaks in toluene-d$_8$.

S5. ^{1}H NMR spectrum of the polyrotaxane **4b** copolymer

The ^{1}H NMR analysis was used to determine the coverage of the rotaxane with macrocycle, *i.e.*, the average number of Psγ-CD macrocycles per repeating unit, which has been calculated using the ratio of the integrated area of the peak assigned to the aromatic protons labeled “l, m, n” in Figure S3 (7.78-7.71 ppm, I_{l+m+n}) and the anomeric H-1 proton of Psγ-CD (5.28 ppm, $I_{H}{}^{1}$); (I H^{l+m+n}6)/(I H^1/8). The average number of PsγCD macrocycles per monomer **1** repeat unit has been found to be 0.37 (*i.e.,* 37.6% coverage).

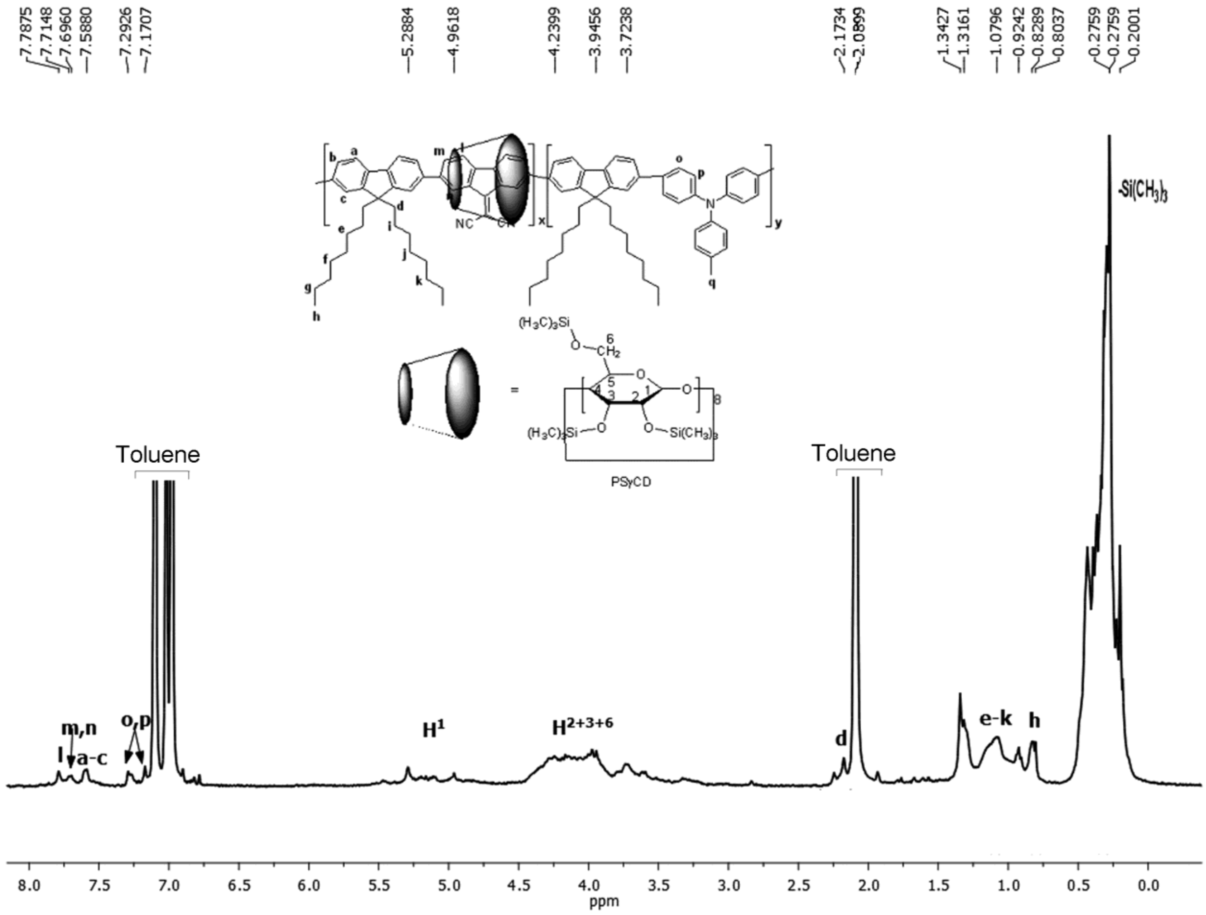

Figure S5: ^{1}H NMR spectrum of the polyrotaxane **4b** copolymer in toluene-d_8 with assignments of the resonance peaks.

S6. The fluorescence lifetime of the non-rotaxane **4** counterpart

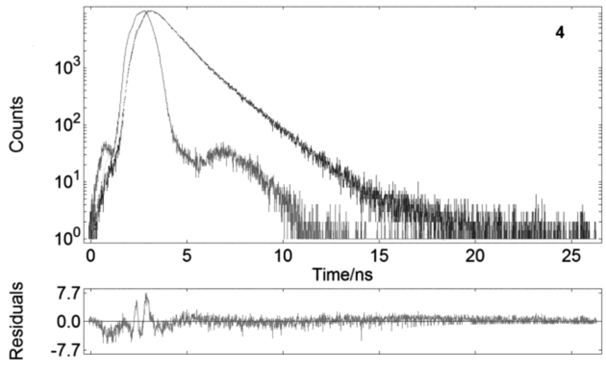

Figure S6: Decay traces of **4** non-rotaxane counterpart.

S7. The fluorescence lifetime of the polyrotaxane **4b** copolymer

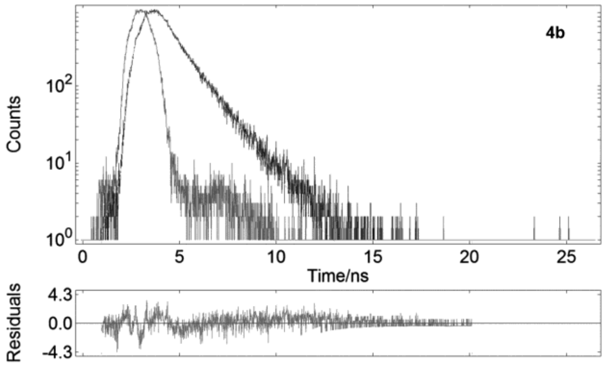

Figure S7: Decay traces of the polyrotaxane **4b** copolymer.

S8. The diagram with HOMO/LUMO levels of the copolymers in addition to the work function of ITO coated glass substrates with PEDOT:PSS (anode) and Ca or Al (cathode)

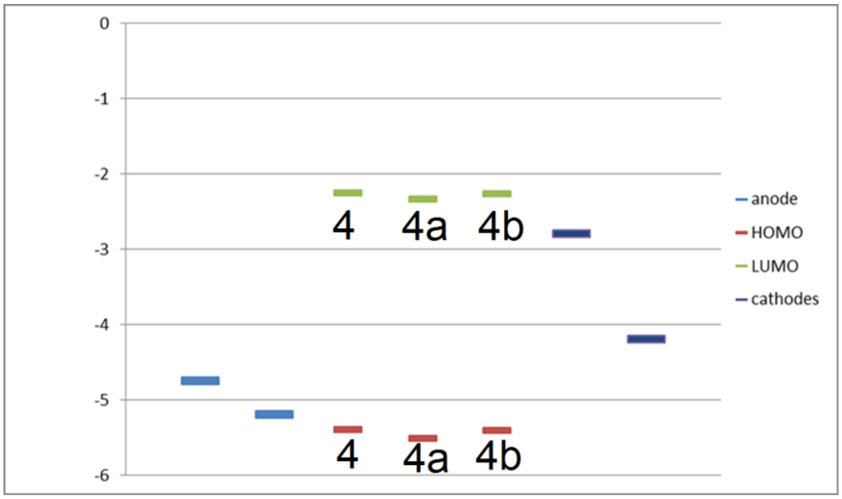

Figure S8: HOMO (red)/LUMO (green) energetic levels in addition to the work function of ITO/PEDOT:PSS (anode) and Ca or Al (cathode).

The diagram with HOMO (red color)/LUMO (green color) levels of the copolymers in addition to the work function of the indium tin oxide (ITO) coated glass substrates with poly(3,4 ethylenedioxythiophene):poly(styrenesulfonate) (PEDOT:PSS) (anode) and Ca or Al (cathode) indicates that the compounds can be suitable for hole and electron transport (HTL) into the PLED active layer [2].

S9. Reference

[1]. Farcas, A.; Janietz, S.; Harabagiu, V.; Guegan, P.; Aubert, P.-H. *J. Polym. Sci. Part A: Polym. Chem*. **2013**, *51*, 1672-1683.

[2]. Al-Ibrahim, M.; Roth, H. K.; Schroedner, M.; Konkin, A.; Zhokhavets, U.; Gobsch, G.; Scharff, P.; Sensfuss, S. *Org. Electron.* **2005**, *6*, 65-77.
